# Response to: Failure of the glymphatic system by increases of jugular resistance as possible link between asthma and dementia

**DOI:** 10.1093/braincomms/fcae038

**Published:** 2024-02-13

**Authors:** Ajay Kumar Nair, Barbara B Bendlin, Douglas C Dean, Melissa A Rosenkranz

**Affiliations:** Center for Healthy Minds, University of Wisconsin-Madison, Madison, WI, 53703USA; Institute on Aging, University of Wisconsin-Madison, Madison, WI, 53706USA; Wisconsin Alzheimer’s Disease Research Center, School of Medicine and Public Health, University of Wisconsin-Madison, Madison, WI, 53792USA; Department of Medicine, School of Medicine and Public Health, University of Wisconsin-Madison, Madison, WI, 53792USA; Wisconsin Alzheimer’s Institute, School of Medicine and Public Health, University of Wisconsin-Madison, Madison, WI, 53792USA; Department of Pediatrics, University of Wisconsin School of Medicine and Public Health, Madison, WI, 53792USA; Department of Medical Physics, University of Wisconsin School of Medicine and Public Health, Madison, WI, 53705USA; Waisman Center, University of Wisconsin-Madison, Madison, WI, 53705USA; Center for Healthy Minds, University of Wisconsin-Madison, Madison, WI, 53703USA; Department of Psychiatry, University of Wisconsin-Madison, Madison, WI, 53719USA

In response to our recent article in *Brain Communications*,^1^ Gallina and colleagues^2^ point to the growing literature on the glymphatic system and offer several testable hypotheses that explore mechanisms linking asthma with poorer brain microstructure, accelerated neuropathology and cognitive decline. Their premise is that our findings could potentially be explained by glymphatic system dysfunction leading to a cascade of poorer waste clearance, higher protein aggregation and progression to dementia.^3^ The suggestions offered by Gallina *et al*. are compelling and we respond to each below. We also point to alternative mechanisms underlying the adverse effects of asthma on the brain, including some proposed in our original article.

## Altered CSF transport in asthma

Gallina *et al*. suggest that ‘detection of a higher number of perivascular spaces in the asthmatic patients’ might suggest a dysfunctional glymphatic system. This is an interesting hypothesis that follows from reports that the number of enlarged perivascular spaces detectable on MR images increases with age, hypertension and inflammation and contributes to dementia risk.^4^ However, this pattern is not associated with cognitive dysfunction among individuals without cognitive impairment or those who are amyloid-β negative.^5^ As our study participants were predominantly cognitively unimpaired and amyloid-β negative, this metric may not be sensitive to potential alterations in the glymphatic system in our sample. An alternative approach that appears to be sensitive to changes in cognitive function and reflects CSF transport more closely is to examine diffusivity along the perivascular space using diffusion tensor imaging.^6^ Though this method is controversial, due to suboptimal sensitivity and specificity,^7^ it may be worth exploring this or related methods in a future study.

## Altered extra- and intra-cranial haemodynamics in asthma

Gallina and colleagues offer the plausible postulation that alterations in CSF flow might be caused by alterations in perivascular flow following resistance in the arteries and veins in the brain, itself driven by increased resistance in systemic circulation. Although this is not possible to test in our sample, we envisage future studies that could directly test this hypothesis among individuals with asthma by examining cerebral haemodynamics using methods such as 4D flow MRI which have revealed age-related decreases in blood flow and increases in pulsatility among cognitively unimpaired individuals, as well as altered metrics of flow and pulsatility among individuals with Alzheimer’s disease dementia.^8^

## Altered cardiovascular health in asthma and association with dementia

In their letter, Gallina *et al*. call for examination of associations between asthma, chronic pulmonary heart disease and dementia. We have pursued this line of inquiry to some extent. There is considerable evidence that asthma has systemic cardiovascular effects, though the underlying mechanisms may differ by asthma phenotype and this remains an area of active research.^9^ We found that individuals with severe asthma have higher concentration of CSF markers of synaptic degeneration.^10^ Furthermore, individuals with severe asthma who had higher levels of cardiovascular risk showed greater elevations in these dementia-relevant biomarkers.^10^ Thus, we report evidence of a link between asthma, cardiovascular health and dementia but also acknowledge the need for replication in other samples.

## Astroglial dysfunction and post-mortem evaluation of aquaporin pathology

Gallina and colleagues point to the literature showing that aquaporin-4 is implicated in glymphatic dysfunction since exchange of cerebrospinal fluid and interstitial fluid depends, in part, on the expression of this passive water transport channel on astrocytic vascular endfeet. This is intriguing as it may connect the data reported in our current article with some of our prior findings. We recently reported that in individuals with asthma, higher plasma concentrations of glial fibrillary acidic protein (GFAP), a marker of reactive astrocytes, were associated with microstructural brain changes associated with deterioration in brain health, such as reduced neurite density (a marker of myelinated axons) across whole brain white matter.^11^ Furthermore, we recently combined RNA sequencing of cells in bronchoalveolar lavage fluid and task-based functional MRI to show that segmental bronchial provocation with allergen, in individuals with mild asthma, is linked to upregulation of genes related to Th17-type inflammation, angiogenesis and astrogliosis, as well as an increase in salience network reactivity, thereby contributing to adverse emotional and cognitive outcomes.^12^ We plan to further explore evidence of astroglial dysfunction in ongoing RNA sequencing analyses of post-mortem brain tissues from individuals with asthma.

## Alternative mechanisms

Apart from these intriguing possibilities advanced by Gallina and colleagues, which are collectively based on the glymphatic dysfunction hypothesis, there are multiple other upstream mechanisms through which asthma might impact brain health. In our article, we point to a few possible pathways, such as neuroimmune interactions via cytokine signalling, increased blood–brain barrier permeability, vascular dysfunction and hypoxia. Some of these pathways have been recently reviewed.^13,14^ Although our discussion so far has focused on the impact of asthma on the brain, the communication is bidirectional such that the impacts could compound. For example, we recently showed that stress-induced activity in the amygdala predicts increases in proinflammatory signalling in the airway and that baseline levels of airway inflammation predict increases in the amygdala response to stress.^15^ Thus, it is likely that multiple pathways collectively contribute to the systemic impact of airway inflammation on the brain, including possible glymphatic dysfunction.

## Competing interests

The authors report no competing interests.

## Data Availability

Data sharing is not applicable to this article as no new data were created or analysed.
